# Micafungin induced apoptosis in *Candida parapsilosis* independent of its susceptibility to micafungin

**DOI:** 10.15698/mic2015.11.236

**Published:** 2015-10-23

**Authors:** Fazal Shirazi, Russel E. Lewis, Dimitrios P. Kontoyiannis

**Affiliations:** 1 Department of Infectious Diseases, Infection Control and Employee Health, The University of Texas M D Anderson Cancer Center, Houston, TX, USA.; 2 Current Address: Department of Medical and Surgical Sciences, University of Bologna, Bologna, Italy.

**Keywords:** apoptosis, micafungin, metacaspase, reactive oxygen species

## Abstract

We hypothesized that the cell wall inhibitor micafungin (MICA) induces apoptosis in both MICA-susceptible (MICA-S) and MICA-non-susceptible (MICA-NS) *Candida parapsilosis*. Antifungal activity and apoptosis were analyzed in MICA-S and MICA-NS *C. parapsilosis* strains following exposure to micafungin for 3 h at 37°C in RPMI 1640 medium. Apoptosis was characterized by detecting phosphatidylserine externalization (PS), plasma membrane integrity, reactive oxygen species (ROS) generation, mitochondrial membrane potential changes, adenosine triphosphate (ATP) release, and caspase-like activity. Apoptosis was detected in MICA exposed (0.25 to 1 mg/L) susceptible *C. parapsilosis* strains and was associated with apoptosis of 20-52% of analyzed cells versus only 5-30% of apoptosis in MICA-NS cells exposed to micafungin (0.5 to 2 mg/L; P = 0.001). The MICA antifungal activity was correlated with apoptotic cells showing increased dihydrorhodamine-123 staining (indicating ROS production), Rh-123 staining (decreased mitochondrial membrane potential), elevated ATP, and increased metacaspase activity. In conclusion, MICA is pro-apoptotic in MICA-S cells, but still exerts apoptotic effects in MICA -NS *C. parapsilosis*.

## INTRODUCTION

*Candida parapsilosis *is a common cause of invasive candidiasis, especially in the setting of catheter associated blood stream infection 1,2,3. The ability of *Candida *spp. to form biofilms on catheters has made candidiasis difficult to treat due to increased resistance to antifungal agents 4. Echinocandins play a significant role in the treatment of invasive candidiasis 5. These agents are non-toxic and exert potent fungicidal activity against *Candida *spp. through the inhibition of (1,3)-β-D glucan synthesis, a major constituent of the fungal cell wall 6. In addition, they are the most promising antifungal agents for lock therapy strategies 4. Although the effects of echinocandin activity have been extensively investigated by standard microbiological endpoints, an improved understanding of the mechanistic basis of micafungin (MICA)-induced cell death in *Candida* may provide new insights into effective antifungal strategies, especially in the era of increasing echinocandin resistance in *Candida* species.

A series of morphologic and biochemical features like exposure of phosphatidylserine (PS), DNA fragmentation, reactive oxygen species (ROS) accumulation and mitochondrial depolarization set apoptosis apart from necrosis 1. Apoptotic responses have been studied in many higher eukaryotes but also have been observed in lower eukaryotes, including yeasts and filamentous fungi 7,8,9,10. As other lower eukaryotes, *Candida *spp*. *exhibit apoptotic markers that are similar to mammalian cells, including externalization of phosphatidylserine, reactive oxygen species (ROS) accumulation, decreased mitochondrial membrane potential, and DNA condensation and fragmentation 3,11. Furthermore, apoptosis can be induced in *C. albicans *by oxidative stress, intracellular acidification and antifungal agents such as amphotericin B and caspofungin 1,12,13,14,15. However, it is unclear whether apoptotic effects in *Candida* are related to the degree of echinocandin susceptibility. Therefore, we hypothesized that the echinocandin MICA, although more pro-apoptotic in micafungin-susceptible (MICA-S) *C. parapsilosis* cells, would also induce apoptosis in micafungin -non-susceptible (MICA-NS) *C. parapsilosis* cells.

## RESULTS

### Micafungin induces more pronounced apoptosis in MICA-susceptible *C. parapsilosis* planktonic cells versus a non-susceptible strain.

The MICA concentrations used for apoptotic studies were based on the susceptibility of the two *C. parapsilosis *isolates to MICA, minimum inhibitory concentration (MIC) for MICA-S strain being 1 mg/L and for MICA-NS 2 mg/L. To assess whether MICA activity occurs through induction of apoptosis, we exposed *C. parapsilosis* planktonic cells to MICA concentration at MIC or sub-MIC level, as it was reported that concentration at MIC or above MIC leads to necrosis, hence annexin-V and propidium iodide (PI) staining were performed following exposure to different concentrations of MICA. For the MICA-S *C. parapsilosis* strain*,* up to 52% of planktonic cells exposed to micafungin (Table 1) showed apoptosis, whereas for the MICA-NS *C. parapsilosis* strain*,* only 30% of MICA - treated planktonic cells were apoptotic (P = 0.001) (Table 1).

**Table 1:** Apoptotic cells and fold changes in fluorescence intensity of MICA-S and MICA-NS C. parapsilosis strains treated with micafungin. ‘-’ Not detected (0% of cells showed particular apoptotic marker). PI, propidium iodide; ROS, reactive oxygen species; ΔΨm, mitochondrial membrane potential; NAC, N-acetyl cysteine.

**Strains**	**Apoptotic Cells**					
	**Protoplast %**		**Fold change**			
***C. parapsilosis***	**Annexin V**	**PI**	**ROS**		**ΔΨ_m_**	
			**-NAC**	**+NAC**	**-NAC**	**+NAC**
**Isolate-1 (MICA-S)**						
Control	5.0 ± 0.01	-	-	-	-	-
0.25 mg/L	24.0 ± 2.0	-	1.8 ± 0.01	1.5 ± 1.00	2.8 ± 0.1	1.9 ± 0.01
0.5 mg/L	52.0 ± 3.0	2.0 ± 0.01	2.4 ± 0.2	1.68 ± 0.20	3.8 ± 0.4	2.48 ± 0.01
1.0 mg/L	19.0 ± 1.0	9.0 ± 1.0	1.9 ± 0.01	1.61 ± 0.21	2.5 ± 0.2	1.8 ± 0.01
**Isolate-6 (MICA-NS)**						
Control	2.0 ± 0.01	1.0 ± 0.01	-	-	-	-
0.5 mg/L	7.0 ± 1.0	-	1.5 ± 0.01	1.0 ± 0.02	1.9 ± 0.01	1.4 ± 0.10
1.0 mg/L	30.0 ± 2.0	-	1.4 ± 0.01	1.3 ± 0.01	1.98 ± 0.01	0.89 ± 0.01
2.0 mg/L	11.0 ± 1.0	6.0 ± 0.01	1.6 ± 0.01	1.1 ± 0.20	2.0 ± 0.01	1.4 ± 0.16

**Figure 1:**
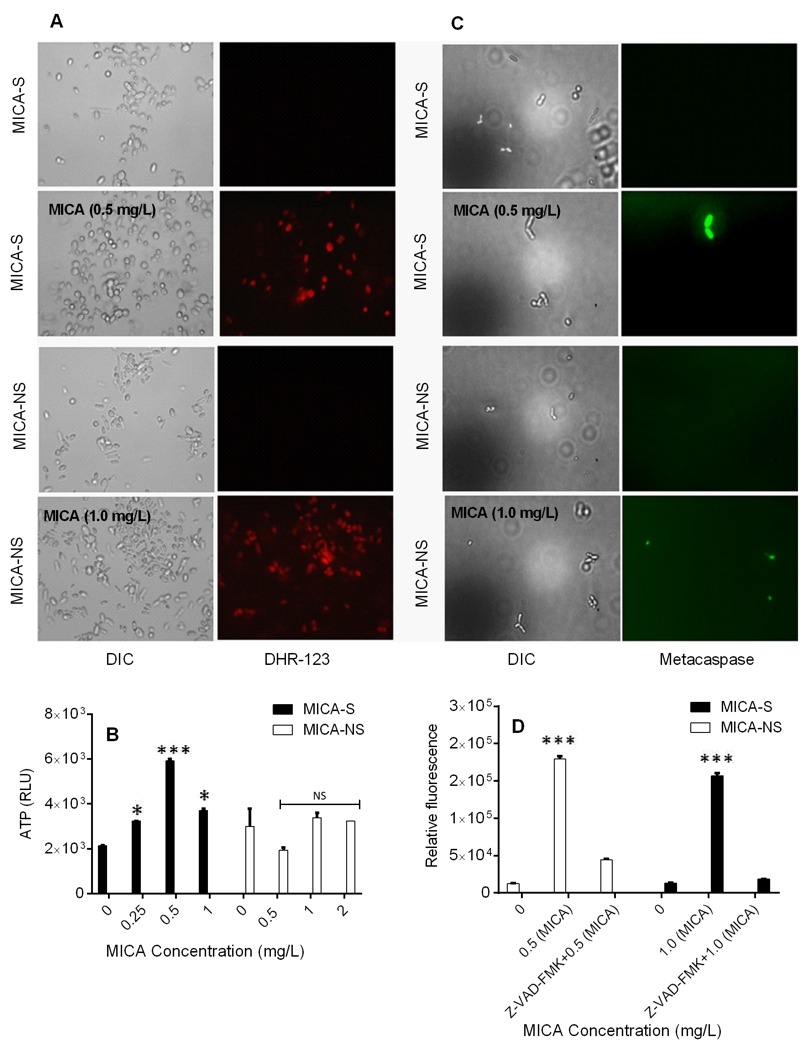
FIGURE 1: Intracellular ROS accumulation, ATP release and activation of caspase like activity in *C. parapsilosis *(MICA-S and MICA-NS isolates 1 and 6, respectively) cells treated with micafungin for 3 h at 37°C. **(A)** Fluorescence images of MICA-S and MICA-NS strains of *C. parapsilosis* treated with micafungin and stained for intracellular ROS with DHR-123. **(B)** ATP release assay indicating *C. parapsilosis* cell membrane disruption and plasma membrane leakage after micafungin treatment. **(C)** Fluorescent images of MICA-S and MICA-NS *C. parapsilosis* strains treated with micafungin and stained with caspase activity detection marker CaspACE FITC-VAD-FMK. **(D)** Relative fluorescence of *C. parapsilosis *MICA-S and MICA-NS cells treated with micafungin with or without caspase-1 inhibitor Z-VAD-FMK, and stained with caspase activity detection marker CaspACE FITC-VAD-FMK. DIC, Differential Interference Contrast; *Cp*, *C. parapsilosis*; RLU, relative light units; *P < 0.05; ***P < 0.0001; NS (non-significant), P > 0.05 (compared with untreated controls).

ROS play an important role as an early initiator of apoptosis in yeasts and other filamentous fungi. MICA-S *C. parapsilosis* planktonic cells exhibited a 2.4 - fold increase in ROS levels compared to untreated cells following MICA exposure. In contrast, a 1.5 fold increase in fluorescence in the MICA-NS *C. parapsilosis* planktonic cell exposed to MICA versus untreated cells (P = 0.006) (Table 1, Fig. 1A). Similarly, MICA exposure was associated with decreases in mitochondrial membrane potential in both MICA-S and MICA-NS strains of *C. parapsilosis* (3.8 fold vs 2.0 fold increase in fluorescence, P = 0.0025, Table 1).

### Micafungin damages the cell membrane of *C. parapsilosis *planktonic cells*, *especially in MICA-S strains

A rapid efflux of ATP due to membrane damage by MICA was detected after the incubation of *C. parapsilosis *planktonic cells with MICA for 3 h at 37°C (Fig. 1 B). Specifically, we observed higher degree of ATP release among MICA-S *C. parapsilosis* strain following MICA exposure (0.5 - 1 mg/L) as compared to MICA-NS strain (Fig. 1B). This increased ATP release was consistent with increased ROS generation and decreased mitochondrial membrane potential of cells incubated with MICA.

**Micafungin** **activates metacaspases (caspase-like activity) in *C. parapsilosis* planktonic cells, especially in MICA-S strains**

Researchers have identified orthologs of mammalian caspases called metacaspases in yeast and filamentous fungi, which are activated in the early stages of apoptosis 8,15. In fungi and plants, metacaspases (caspase-like) activity can be assessed using the *in situ* detection marker CaspACE FITC-VAD-FMK 9,10. To confirm the presence of metacaspase activation, MICA - pretreated (0.5 - 1 mg/L) *C. parapsilosis* planktonic cells were incubated with CaspACE FITC-VAD-FMK, which fluoresces green when it binds to active metacaspases. Indeed, the planktonic cells of MICA-S and MICA-NS* C. parapsilosis* treated with MICA showed green fluorescence, suggesting that apoptosis in these cells occurs via metacaspase activation irrespective of MICA susceptibility (Figure 1 C, D).

To further support the concept that caspase-like activity are involved in apoptosis, we treated* C. parapsilosis* planktonic cells with 0.5 to 1.0 mg/L MICA for 3 h in the presence or absence of the caspase 1 inhibitor Z-VAD-FMK at a concentration of 40 μM. MICA treated cells were assessed for the metacaspase activity in *Z*-VAD-FMK - treated and untreated samples of the *C. parapsilosis* planktonic cells. *C. parapsilosis* susceptible planktonic cells treated with MICA in combination with Z-VAD-FMK exhibited less fluorescence in cells (3.38 fold), than did cells treated with only MICA (13.74 fold). Similarly, *C. parapsilosis* non-susceptible cells showed decrease in fluorescence (1.41 fold) in Z-VAD-FMK - treated cells than in untreated cells (11.8 fold) (Figure 1D).

Finally, to further elucidate the ROS role in apoptosis, we examined whether the ROS scavenger NAC reverses apoptosis in *C. parapsilosis* S and NS planktonic cells. At 37°C, the levels of intracellular ROS were markedly decreased from 2.4 fold to 1.6 fold in susceptible cells and 1.6 to 1.0 fold in non-susceptible cells, after the addition of ROS scavenger *N*-acetyl-cysteine (NAC, 40 mM), compared to NAC unexposed cells (Table 1). Similar observation was evidenced in mitochondrial potential of *C. parapsilosis* S and NS planktonic cells treated with MICA in the presence of NAC (Table 1).

## DISCUSSION

We found that MICA induced cellular apoptosis in both MICA-S and MICA-NS strains. After exposure to sub-inhibitory concentrations of MICA, PS was exposed on the outer surface of the plasma membrane, accompanied by an increase in intra-cellular ROS levels and depolarization of the mitochondrial membrane, which was marked by ATP and cytochrome *c* release. Hao *et al.* 13 reported that caspofungin exerts its antifungal activity against *C. albicans *by apoptosis. In concordance with these reports, our results have described that dying *C. parapsilosis* cells exhibit markers of apoptosis following MICA exposure (0.25 - 2 mg/L). Our results show that apoptosis markers in *C. parapsilosis *were detectable following even sub-MIC MICA concentrations in both MICA-S and MICA-NS strains. In addition, a higher percentage of MICA-S *C. parapsilosis* cells underwent apoptosis compared to MICA-NS strains following MICA exposure. This phenomenon could be attributed to a more complex glucan matrix in the cell walls of the MICA-NS strains, which might account for MICA-NS strains resistance to antifungal drugs, as described previously 16.

Caspases, cysteine-aspartic acid proteases, when cleaved, induce apoptosis. In *C. albicans*, a putative caspase encoded by metacaspase (Ca*MCA1*) has been shown to be involved in apoptosis 12,13. In our study, we used CaspACE FITC-VAD-FMK, which detects activated caspases, to show that *C. parapsilosis *caspases are activated in response to MICA exposure. These data are consistent with the hypothesis that apoptosis of *C. parapsilosis* proceeds through metacaspase-dependent pathways, although further studies are needed to demonstrate how metacaspase activity contributes to this process.

In conclusion, MICA initiate apoptosis of *C. parapsilosis* cells at concentrations below the minimum inhibitory concentration in both MICA-S and MICA-NS strains, although more susceptible and non-susceptible *C. parapsilosis *strains will need to be tested to confirm generalization of our findings. The mechanism of the apoptotic pathways induced by MICA needs further clarification, as do the roles of apoptosis in determining the therapeutic efficacy of these echinocandins *in vivo*. Given the differences in the architecture of the death-regulating machineries of fungi and higher mammals 7, apoptotic pathways may represent important targets for novel antifungal drug development and should be further investigated as a new antifungal approach.

## MATERIALS AND METHODS

### Echinocandins

Pure MICA powder (5 mg/mL stock solution) was obtained from Astellas Pharma US, Inc. The stock dilutions were prepared in distilled water and stored at -80°C until use.

### Isolates and growth conditions

The *C. parapsilosis* isolates used in this study were from blood cultures of cancer patients with candidemia at The University of Texas MD Anderson Cancer Center, Houston, TX. Specifically, we used one MICA-S and one MICA-NS *C. parapsilosis* isolate. The isolates were grown on yeast peptone dextrose (YPD) agar plates overnight at 37°C. The cells were then collected and washed twice in phosphate-buffered saline and counted using a hemocytometer (Hausser Scientific). Next, 10^6^ cells/mL of each isolate were resuspended in liquid YPD and incubated at 37°C for 12 h until reaching mid-log phase, and recounted with a hemocytometer to achieve final testing inocula.

### Susceptibility testing

We performed broth microdilution susceptibility testing according to the Clinical and Laboratory Standards Institute-approved document M27-A3, in 96 well microtitration plates in RPMI 1640 medium (Corning Inc., New York, NY) containing serial two fold dilutions of micafungin and a final inoculum of 5 x 10^3 ^cells/mL of each isolate. The minimum inhibitory concentration (MIC) of MICA for each isolate was determined visually 24 h after incubation at 37°C as the lowest concentration that resulted in a prominent decrease in turbidity (reduction of > 50% of growth) and the results were analyzed according to guidelines set for *Candida* susceptibility to echinocandins 17,18.

### Detection of apoptosis and necrosis with annexin V and propidium iodide (PI) double staining of *C. parapsilosis* cells

The apoptosis marker phosphatidylserine (PS) is located on the inner leaflet of the lipid bilayer of the cytoplasmic membrane, and is translocated to the outer leaflet at the onset of apoptosis, where it can be identified by annexin V staining 9,10,19. PI staining identifies necrotic cells, as it does not permeate cells in intact membranes. Therefore, staining patterns differentiate between live cells (Annexin V-/PI-), cells undergoing early apoptosis (Annexin V+/PI-) and necrotic cells (Annexin V+/PI+) 19,20. Cells treated with micafungin (0.25-2 mg/L), were digested with a lysing enzyme mixture (0.25 mg/mL of chitinase, 15 U of lyticase, and 20 mg/mL lysing enzyme; Sigma-Aldrich) for 3 h at 30°C. After digestion, cells were stained with the annexin V - fluorescein isothiocyanate (FITC) (50 mg/L, BD Pharmingen, USA) and PI (50 mg/L) at room temperature (RT) for 15 min and then observed under a Nikon Microphot SA fluorescence microscope to assess the externalization of the apoptosis marker PS as previously described 9,10.

**Detection of intracellular ROS** **in** ***C. parapsilosis*** **cells**

**Intracellular ROS levels in *C. parapsilosis* cells were measured as previously described 9,10.** *C. parapsilosis* cells were treated with MICA (0.25-2 mg/L) for 3 h at 37°C. Cells were then spiked with dihydrorhodamine (DHR)-123 (5 mg/L). After incubation for 2 h at RT, cells were harvested after centrifugation at 13,000 x *g* for 5 min and observed with a Nikon Microphot SA fluorescence microscope (excitation, 488 nm; emission, 520 nm). For quantitative assays, fluorescence intensity values were recorded using a POLARstar Galaxy microplate reader (excitation, 490 nm; emission, 590 nm; BMG LABTECH, Offenburg, Germany). The same experiment was conducted in the presence of NAC at a concentration of 40 mM.

### Mitochondrial membrane potential (ΔΨ_m_) measurements in *C. parapsilosis* cells

Mitochondrial membrane depolarization was assessed by staining with rhodamine (Rh)-123, a fluorescent dye that is distributed in the mitochondrial matrix as previously described 9,10. Briefly, the cells were exposed to micafungin (0.25-2 mg/L) for 3 h at 37°C and harvested via centrifugation, washed twice, then resuspended in phosphate-buffered saline. Rh-123 was added to the final concentration of 10 μM and was incubated for 30 min in the dark at RT. Fluorescence intensity was recorded as described above. The same experiment was conducted in the presence of NAC at a concentration of 40 mM.

### Adenosine triphosphate (ATP) release assay in *C. parapsilosis* cells

We assessed the severity of MICA-induced mitochondrial and plasma membrane damage by the amount of cellular ATP released into the medium as described by Ben-Ami *et al.* 21. The *C. parapsilosis* cells were then counted with a hemocytometer (Hausser Scientific), and suspended in RPMI 1640 medium at 10^6^ cells/mL. After 12 h at 37°C, the medium was removed by centrifugation at 13,000 x *g* for five min and cells were re-suspended in MICA-containing (0.25 - 2 mg/L) or drug-free RPMI 1640 medium. After 3 h of incubation, the cells were removed by centrifugation as described above and the ATP released in the supernatants was assayed by using the CellTiter-Glo luminescent kit (Promega). Data were recorded with a microplate luminometer (SpectraMax M5; Molecular Devices).

**Detection of metacaspase activity** **in *C. parapsilosis* cells**

Activation of metacaspases was detected with the CaspACE FITC-VAD-FMK *in situ* Marker (Promega) 9,10. The cells were pretreated with MIC (0.5 - 1 mg/L) for 3 h at 37°C. Cells were harvested, washed in phosphate-buffered saline, and then re-suspended in 10 μM CaspACE FITC-VAD-FMK solution. After 2 h of incubation at RT, cells were washed twice and re-suspended in phosphate-buffered saline. Samples were mounted and viewed in a Nikon Microphot SA fluorescence microscope (excitation, 488 nm; emission, 520 nm).

Inhibition of apoptosis was performed by incubating *C. parapsilosis *planktonic cells with MICA, in the presence or absence of the caspase-1 inhibitor Z-VAD-FMK (Sigma) and the antioxidant *N*-acetyl-cysteine (NAC) at final concentrations of 40 μM and 40 mM, respectively. After incubation for 3 h, cells were analysed for ROS accumulation, mitochondrial potential and metacaspase activity.

### Statistical analysis

For all assays, three independent experiments were performed on different days in triplicate. Multiple treatment groups were compared using Kruskall-Wallis test and post-hoc paired comparisons were compared using Dunnett’s tests. Calculations were made with InStat (GraphPad Software). All results are expressed as means ± standard deviations. Two-tailed P values of less than 0.05 were considered statistically significant.
